# Association between Large Neutral Amino Acids and Brain Integrity in Middle-Aged Adults at Metabolic Risk

**DOI:** 10.21203/rs.3.rs-3951968/v1

**Published:** 2024-02-16

**Authors:** Cherry Youn, Marie L. Caillaud, Yanrong Li, Isabelle A. Gallagher, Barbara Strasser, Dietmar Fuchs, Hirofumi Tanaka, Andreana P. Haley

**Affiliations:** a Department of Psychology, The University of Texas at Austin, Austin, Texas, USA; b Medical Faculty, Sigmund Freud Private University Vienna, Vienna, Austria; c Institute of Biological Chemistry, Biocentre, Medical University of Innsbruck, Innsbruck, Austria; d Department of Kinesiology and Health Education, The University of Texas at Austin, Austin, Texas, USA

**Keywords:** White matter hyperintensity, Metabolic Syndrome, Phenylalanine, Midlife Adults

## Abstract

This investigation delves into the interplay between large neutral amino acids (LNAA) and metabolic syndrome (MetS) in midlife adults, examining their collective influence on brain structure and cognitive function. While LNAA, such as tryptophan and phenylalanine, are known to bolster cognition in youth, our study hypothesizes a reversal of these benefits in older adults with MetS, potentially signaling premature cognitive aging. Eighty participants between 40–61 years underwent MetS component quantification, LNAA measurement via high-performance liquid chromatography, and brain imaging to evaluate white matter hyperintensity (WMH) volume and medial temporal lobe (MTL) cortical thickness. Our linear regression analysis, adjusting for sex, age, and education, revealed that phenylalanine levels moderated the relationship between MetS and WMH volume (*F*(6, 69) = 3.134, *p* < 0.05, *R*^2^ = 0.214), suggesting that MetS’s cognitive impact may be partly due to phenylalanine catabolism byproducts. However, LNAA metabolites did not significantly modulate the MetS-MTL cortical thickness relationship. The findings suggest that LNAA metabolic dysregulation, marked by elevated levels in the presence of MetS, could correlate with brain structural compromises, particularly in the form of MTL cortical thinning and increased WMH load, detectable in midlife. This nuanced understanding of LNAA’s role in cognitive health amid cardiovascular risk factors is pivotal, proposing a potential biomarker for early intervention. Further research is crucial to elucidate the longitudinal influence of LNAA and MetS on brain health, thereby informing strategies to mitigate cognitive decline.

## Introduction

The intricate interplay between brain health and cardiovascular risk has garnered significant attention in brain imaging and behavior research (Friedman et al., 2014; Marebwa et al., 2018). In particular, the transition from midlife to old adulthood marks a critical phase in which the convergence of aging and cardiovascular diseases can exert profound effects on neural integrity and cognitive function (United Nations, 2022). The cumulative effects arising from multiple sub-clinical elevations in cardiovascular risk factors have been shown to yield increased predictive value for cognitive impairment above and beyond the sum of individual conditions (Segura et al., 2009a; Solfrizzi et al., 2010). This cluster of risk factors is known as metabolic syndrome (MetS) (Eckel et al., 2005). Cognitive impairment in individuals with metabolic risk may stem from cerebrovascular disease and subsequent alterations in white matter integrity (Segura et al., 2009a; Segura et al., 2009b). White matter hyperintensities (WMH) are macrostructural lesions often of vascular origin and are significant indicators of cognitive decline and are positively associated with MetS components (Dufouil et al., 2001; Jagust et al., 2005; Tiehuis et al., 2014). Additionally, brain tissue alterations in gray matter (e.g., cortical thickness) have also been linked to cognitive function (Dickerson et al., 2008) and MetS components (Hassenstab et al., 2012; Leritz et al., 2011). In particular, MetS is associated with reduced cortical thickness in medial temporal lobe (MTL; McIntosh et al., 2017), a region intimately connected with memory consolidation and retrieval. Given MTL’s critical role in memory, middle-aged adults with MetS may be at heightened risk for accelerated cerebral atrophy in this area, which could presage cognitive decline and increase susceptibility to dementia.

A critical aspect in alleviating the adverse brain and cognitive effects of MetS involves a clear understanding of the role of nutrition. Large neutral amino acids (LNAA), such as tryptophan and phenylalanine, are essential amino acids that play a significant role in the regulation of MetS components (Oxenkrug, 2010; Strasser, 2017). Investigations into dietary intake and supplementation of LNAA have suggested potential links to MetS components and outcomes, where dietary tryptophan consumption was inversely associated with MetS (Wang et al., 2021). In animal models, tryptophan supplementation improved blood pressure regulation and glucose metabolism in spontaneously hypertensive rats (Ardiansyah et al., 2011). In direct contradiction to these findings, a positive association between serum tryptophan levels and MetS components has been observed (Chen et al., 2016). Additionally, there is evidence of a positive association between serum levels of both tryptophan and phenylalanine and type 2 diabetes (Chen et al., 2016; Wang et al., 2011). These nuanced findings underscore the complexity of LNAA effects, which may vary considerably with age-related variations in metabolism.

The association between LNAA with cognitive processes adds a layer of complexity since changes in tryptophan and phenylalanine play a role in cognitive decline (Flacker & Lipsitz, 2000). Variations in brain tryptophan and phenylalanine concentrations modify the synthesis and release of serotonin and catecholamines, respectively. The exploration of the cognitive effects of phenylalanine and tryptophan remains mostly focused on individuals with phenylketonuria or young adults under stress conditions or individuals with psychiatric disorders (van de Rest et al., 2013). Several studies suggest an association between higher serum phenylalanine concentrations and Alzheimer’s disease in late adulthood (Wissmann et al., 2013). Similarly, increased levels of tryptophan have been linked with the pathophysiology of mood disorders, schizophrenia, and Alzheimer’s disease among older populations (Anderson & Maes, 2013).

In light of existing research gaps, this study addresses the need for an understanding of the roles played by tryptophan and phenylalanine, particularly within midlife adults at varying metabolic risks. Neuroimaging offers insight into the physiological mechanisms underlying cognitive function and impairment, facilitating a more comprehensive understanding of the relationship between LNAA, cardiovascular risk factors, and cognitive functioning. Furthermore, this study explores uncharted territory by examining the interaction effects of MetS and LNAA on brain health. Given the documented adverse cognitive effects of LNAA imbalance among older adults and their potential link to MetS onset, this study explores the interactions between MetS and LNAA on cortical thickness and white matter integrity. We hypothesize that (a) an interaction between LNAA metabolic dysregulation, as indicated by higher LNAA levels, and a higher number of MetS components may be associated with compromised brain integrity in terms of cortical thinning in the MTL and increased WMH burden, and (b) these changes can be detected as early as in midlife.

## Methods

### Participants

This study included 80 middle-aged adults (age range: 40–61 years). Participants with a history of neurological disease, major psychiatric illness, previous hospitalization of substance use, or contraindications of MRI were excluded. The study protocol consisted of two visits: a health assessment visit and a neuroimaging visit completed within one month of each other. All participants provided written informed consent for all study procedures, which were approved by the University of Texas at Austin Institutional Review Board (approval number 2011070025).

### Procedure

During the first visit, participants underwent a general health assessment that included blood pressure monitoring and fasted venipuncture blood draw to obtain concentrations of glucose, triglycerides, total cholesterol, and HDL-cholesterol using a standard enzymatic technique. Based on the examination completed during this phase, the number of MetS components (0–5) was calculated according to the unified criteria (Alberti et al., 2009). The categorical cut points for each component include the following: (a) waist circumference > 102 cm in males and > 88 cm in females; (b) triglyceride levels ≥ 150 mg / dL (1.7 mmol/L); (c) HDL-c levels < 40 mg/dL (1.0 mmol/L) in males and < 50 mg/dL (1.3 mmol/L) in females; (d) systolic blood pressure levels ≥ 130/85 mmHg or diastolic blood pressure levels ≥ 85 mmHg; (e) fasting blood glucose levels ≥ 100 mg/dL. Serum concentrations of tryptophan, kynurenine, phenylalanine, and tyrosine were measured using liquid chromatography (Neurauter et al., 2013). The ratios of (a) kynurenine and tryptophan, and (b) phenylalanine and tyrosine were calculated as indexes of tryptophan degradation and phenylalanine 4-hydroxylase (PAH) activity, respectively.

#### Neuroimaging parameters

##### MRI Data acquisition

Structural MRI images of participants were collected during the second visit. MRI acquisitions were performed on a 3.0-T Siemens Skyra scanner (Siemens Medical Solutions, Malvern, PA) equipped with a 32-channel head coil. Anatomical scans (T_1_ weighted images) of the entire brain were collected using high-resolution Magnetization-Prepared Rapid Acquisition Gradient-Echo (MPRAGE) sequences (256 × 256 matrix, flip angle = 7°, field of view = 24 × 24 cm^**2**^, 1-mm slice thickness, 0 gap, voxel size = 1.0 × 1.0 × 1.0 mm^**3**^, repetition time = 2530.0 ms). The imaging protocol also included a T_2_ image using FLAIR sequence (axial plane, echo time k9TE) = 75 ms, repetition time (TR) = 9500 ms, field of view (FOV) = 24 × 24 mm^**2**^, 42 slices, 3 mm slice thickness, 0.3 gap).

##### MRI Data Processing

The MPRAGE images were processed using the Freesurfer Imaging Analysis Suite, an open-source package freely available for download to analyze and visualize neuroimaging data (http://surfer.nmr.mgh.harvard.edu). The process of cortical reconstruction and volumetric segmentation includes motion correction and averaging (Reuter et al., 2010) of 2 volumetric weighted images. The subsequent steps involve computerized removal of non-brain tissue using a hybrid watershed/surface deformation procedure (Ségonne et al., 2004), followed by automated Talairach transformation and intensity normalization. The gray matter-white matter boundary is then tessellated, and any automated topological defects are corrected automatically (Fischl et al., 2001; Ségonne et al., 2007). Finally, surface deformation occurs following intensity gradients, which optimally places the gray/white and gray/cerebrospinal fluid borders at the points of greatest shift, marking the transition between tissue classes (Dale et al., 1999; Fischl & Dale, 2000).

FLAIR images were processed using the Lesion Segmentation Tool version 1.2.3, an automated algorithm in Statistical Parametric Mapping 8. Based on spatial and intensity probabilities from T_1_ images and hyperintensity outliers on T_2_ FLAIR images, we assigned voxels to tissue probability maps and gave them a probability of being a white matter lesion. We applied an initial threshold of 0.3 to create lesion seeds, and it was used to generate the conservative lesion belief map from the gray and white matter voxels. Next, a growth algorithm grew these seeds towards a liberal lesion belief map that contained gray matter, white matter, and cerebrospinal fluid lesion belief maps. Finally, we used a threshold of 0.99 on the resulting lesion belief map to remove any voxels with a lower probability of being a lesion. The resulting total WMH volume was divided by intracranial volume and multiplied by 100 to give a WMH ratio in units of percentage of intracranial volume.

##### MRI Analyses

Cortical thickness in the temporal lobe subregions were extracted and analyzed. Using the Desikan-Killiany atlas (Desikan et al, 2006), this study delineated the boundaries of the following regions of interest: superior, middle, and inferior temporal gyri, banks of the superior temporal sulcus, fusiform gyrus, transverse temporal gyrus, entorhinal cortex, temporal pole, and parahippocampal cortex and average them to create an MTL composite score. This approach is substantiated by findings that link MetS and cortical thinning in the MTL (Bakkour et al., 2009; McIntosh et al., 2017). We calculated the ratio of whole-brain WMH to total intracranial volume (TIV), as determined by Freesurfer, and applied a natural logarithmic transformation to normalize the distribution of the variable for subsequent analyses.

### Statistical Analyses

This study examined the main effects of LNAA levels and a number of MetS components on brain integrity using multiple linear regression analyses. We tested if tryptophan, kynurenine, tyrosine, and phenylalanine moderate the relationships between MetS and MTL cortical thickness and WMH volume. A natural log transformation was utilized on WMH-to-TIV ratio resulting in the normality of the variable. Sex, age, and years of education were included as covariates to control for their potential confounding effects on brain integrity and cognitive performance. All results were analyzed using the R statistical software package (R Core Team, 2021).

## Results

As described in Table 1, the participants were middle-aged, cognitively unimpaired, ethnically diverse, and representative of the population of the state of Texas. The serum concentrations of kynurenine, tryptophan, phenylalanine, and tyrosine were within normal levels of LNAA although the mean tyrosine level was slightly higher (>75^th^ percentile), and the mean tryptophan level was lower (~25^th^ percentile) than a cohort of healthy blood donors (Geisler et al., 2015). Table 2 summarizes intercorrelations among all variables of interest. The number of MetS components was significantly correlated with all LNAA except for tryptophan. The MTL composite was negatively and significantly associated with WMH volume (r = −.41, *p* < .001). Aside from the expected correlations among the metabolites and their ratios, tyrosine was positively and significantly associated with kynurenine (r = .24, *p* < .05).

**Figure 1.**
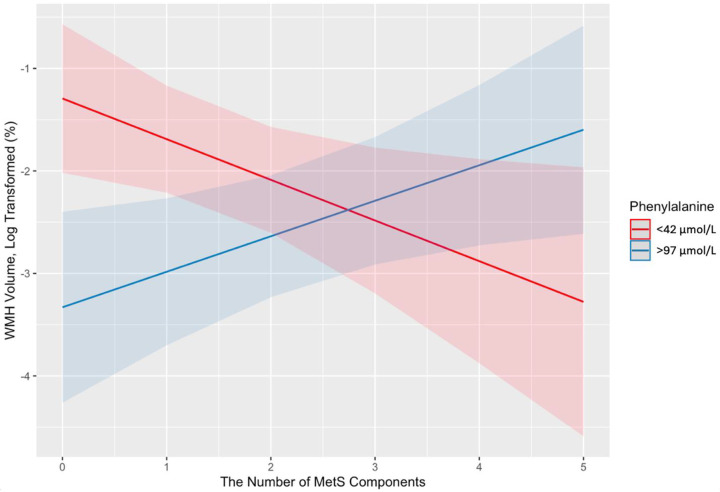
The interaction of the number of MetS components and phenylalanine on WMH volume. Phenylalanine level is categorized into two groups, including (a) 2 standard deviations below the mean, and (b) 2 standard deviations above the mean. The shaded areas surrounding the lines represent the 95% confidence intervals. MetS = metabolic syndrome; WMH = white matter hyperintensity.

We first examined LNAA metabolites as moderators of the effect of MetS on WMH. Controlling for age, sex, and years of education, phenylalanine moderated the relation between the number of MetS components and WMH volume (*β* = 0.01, *SE* = 0.01, *p* = .002). Figure 1 shows the predicted values of WMH across varying levels of MetS with lower and higher phenylalanine levels (two standard deviations below and above the sample mean). At lower levels of MetS components (0 or 1), we observe a significant difference in WMH volume between participants with lower versus higher phenylalanine levels. However, as the level of MetS increases, we observe the opposite effect on WMH between the two phenylalanine groups, and the predicted difference becomes nonsignificant. We observed no significant interactions when testing LNAA metabolites as moderators of MTL composite. However, upon exploratory follow-up analyses, each LNAA metabolite showed significant moderation effects on the association between the number of MetS components and thickness measurements within different subregions of the MTL. More specifically, phenylalanine moderated the relation between the number of MetS components and cortical thickness in the right fusiform gyrus (*β* = −0.001, *SE* = 0.001, *p* = .04) and transverse temporal gyrus (*β* = 0.002, *SE* = 0.001, *p* = .03). Tyrosine moderated the relationship between the number of MetS components and cortical thickness in the left banks of the superior temporal sulcus (*β* = −0.001, *SE* = 0.000, *p* = .04) and parahippocampal cortex (*β* = −0.001, *SE* = 0.001, *p* = .04). Similarly, kynurenine moderated the relationship between the number of MetS components and cortical thickness in the right banks of the superior temporal sulcus (*β* = 0.035, *SE* = 0.017, *p* = .04). Lastly, tryptophan moderated the relationship between the number of MetS components and cortical thickness in the left temporal pole (*β* = −0.005, *SE* = 0.022, *p* = .03).

## Discussion

Using a sample of middle-aged adults at varying levels of metabolic risks, we found associations between LNAA, brain integrity, and the accumulation of MetS components. More specifically, phenylalanine level was a significant moderator of the association between the number of MetS components and WMH volume. Higher serum phenylalanine levels were associated with a reduced volume of WMH in individuals with a low count of MetS components, while higher WMH volume was observed in those with high numbers of MetS components. The interaction remained significant even after controlling for key sociodemographic covariates. There is limited literature exploring the relationship between phenylalanine levels and WMH among healthy adults, as the research focus has been primarily on individuals with PKU. Our findings show an interesting pattern, where we observed the effects of MetS on WMH to be in opposite directions between high and low phenylalanine groups. These opposing trends suggest that phenylalanine may play a differential role in the progression of cerebral white matter changes depending on its concentration in the context of MetS. The finding of high phenylalanine being related to a greater volume of WMH in adults with MetS is consistent with literature relating high hydroxyphenylpyruvic acid, a potentially toxic compound derived from phenylalanine catabolism, and evidence linking higher hydroxyphenylpyruvate to greater WMH volumes in a large sample of middle-aged and older adults (Sliz et al., 2022). Elevated hydroxyphenylpyruvic acid is characterized by increased activities of enzymes involved in tyrosine metabolism (Civen & Brown, 1971). Moreover, higher levels of phenylalanine and tyrosine have been associated with an increased risk of cardiovascular disease (Würtz et al., 2012; Würtz et al., 2015), but the effect of phenylalanine on structural brain changes among individuals at metabolic risks was not examined in those studies. However, the mechanism that might link higher concentrations of phenylalanine to lower WMH volumes in adults without MetS is unclear. Thus, further research is warranted to elucidate the interplay among phenylalanine, metabolic risk, and WMH.

Our analysis did not reveal significant moderating effects of LNAA metabolites on the relationship between MetS and cortical thickness in our composite measure of the MTL region. The lack of significance may be attributed to the complexity of MetS’s impact on cortical thickness, as its various components may influence cortical thickness in different ways. While some studies have reported cortical thinning in individuals with MetS (Lu et al., 2021; Song et al., 2015), others have reported higher cortical thickness may be associated with some subcomponents of MetS such as higher cholesterol (Leritz et al., 2011) and higher visceral adiposity (Cho et al., 2021; Kaur et al., 2015). It is also possible that our study has not captured the full extent of the interaction between the number of MetS components and cortical thickness, particularly at elevated LNAA levels. This could stem from regional variations within the MTL composite measure that may dilute the detection of specific effects. Consequently, the lack of a significant differentiated impact of LNAAs on cortical thickness across varying MetS components suggests that the composite measure may not adequately capture the nuanced regional influences. Future research should, therefore, focus on the individual MetS component of cortical structure as well as examining regional variation within the MTL.

Our findings should be interpreted in light of the study’s limitations. First, our sample lacks data on additional metabolites (e.g., hydroxyphenylpyruvic acid) that may explain the moderating effects between the number of MetS components and neural integrity. Second, power is often a limitation for detecting interaction effects (McClelland & Judd, 1993) and we were limited by a modest sample size. Thus, our findings must be replicated and cross-validated in a larger sample with a wider range of ages. Nonetheless, our findings support linkages between LNAA and neural integrity in midlife adults at metabolic risks.

Behavioral factors play a significant role in the development and progression of MetS. Predominant among these is the adherence to health-related behaviors. Unfavorable dietary habits, which are prevalent among midlife adults (Wang et al., 2014), contribute to the pathogenesis of both cardiovascular and neurodegenerative diseases, including dementia (Livingston et al., 2020). Addressing these lifestyle factors, particularly through optimizing nutrition, is poised to be a pivotal strategy in reducing the risk of MetS and its associated cognitive decline (Power et al., 2020). Our study contributes to the field of nutritional neuroscience by investigating the associations between LNAA levels and brain health. Our results suggests that a stable, health-conscious diet in midlife could be crucial in mitigating accelerated cognitive aging associated with MetS.

## Conclusions

The present study underscores the modulatory role of LNAA, particularly phenylalanine, on the relationship between MetS and structural brain changes during midlife. Specifically, phenylalanine levels influence the extent of WMH in relation to the number of MetS components, even after adjusting for relevant covariates. Our findings enrich our understanding of the neurological implications of MetS in midlife, opening avenues for lifestyle interventions aimed at mitigating the risk of cognitive decline through the modulation of LNAA pathways.

## Figures and Tables

**Table 1.** Participant Characteristics (*n* = 80)

Participant Characteristics	Mean ± SD
Demographic Characteristics	
Age, years	50.2 ± 6.5
Male/Female, n	35/45
Education, years	15.6 ± 2.6
Race, *n* (%)	
Non-Hispanic White	43 (54%)
Hispanic	24 (30%)
African American	7 (9%)
Multi-racial	1 (1%)
Other	5 (6%)
LNAA Measures	
Kynurenine, μM	1.78 ± 0.48
Tryptophan (males), μmol/L	60.20 ± 8.68
Tryptophan (females), μmol/L	53.52 ± 9.27
Phenylalanine, μmol/L	69.30 ± 13.63
Tyrosine, μmol/L	121.93 ± 32.77
Metabolic Syndrome Components, *n* (%)	
0	18 (23%)
1	25 (31%)
2	9 (11%)
3	12 (15%)
4	11 (14%)
5	5 (6%)
Neural Integrity	
Medial Temporal Lobe Composite (mm^2^)	2.70 ± 0.11
White Matter Hyperintensity, Log Transformed (%)	−2.15 ± 0.99

*Note:* SD = standard deviation; LNAA = large neutral amino acids

**Table 2.** Intercorrelations among measured variables

	Kynu	Tryp	K/T	Phen	Tyro	P/T	MetS	MTL	WMH
**Moderators**									
Kynurenine (Kynu)	1.00	-	-	-	-	-	-	-	-
Tryptophan (Tryp)	**0.39**	1.00	-	-	-	-	-	-	-
Kynurenine/Tryptophan (K/T)	**0.77**	−**0.28**	1.00	-	-	-	-	-	-
Phenylalanine (Phen)	0.19	0.18	0.08	1.00	-	-	-	-	-
Tyrosine (Tyro)	**0.24**	0.30	0.07	**0.57**	1.00	-	-	-	-
Phenylalanine/Tyrosine (P/T)	−0.16	−0.16	−0.06	0.18	−**0.67**	1.00	-	-	-
**Predictor**									
MetS components	**0.31**	−0.03	**0.34**	**0.26**	**0.33**	−0.13	1.00	-	-
**Outcome**									
Medial Temporal Lobe Composite (MTL)	0.10	0.09	0.04	−0.04	−0.07	0.00	−0.16	1.00	-
WMH Volume, Log Transformed	0.00	−0.03	0.03	−0.04	0.01	−0.03	0.02	−**0.41**	1.00

*Note:* MetS = metabolic syndrome; WMH = white matter hyperintensity

## Data Availability

The data that support the findings of the study are available upon request from the corresponding authors [H.T. & A.P.H.] from the MetS Study.
